# Food Away From Home and Self-Perceived Gastrointestinal Health

**DOI:** 10.3389/fnut.2021.741647

**Published:** 2021-11-26

**Authors:** Jyh-Jou Chen, Li-Yun Tsai, Jung-Mei Tsai, Chen-Yuan Hsu

**Affiliations:** ^1^Gastroenterology and Hepatology Department, Chi-Mei Medical Center, Tainan, Taiwan; ^2^College of Nursing and Health Sciences, Dayeh University, Changhua, Taiwan; ^3^Mackay Memorial Hospital, Taipei City, Taiwan; ^4^Department of Nursing, Mackay Junior College of Medicine, Nursing, and Management, Taipei City, Taiwan; ^5^Department of Nursing, Mackay Medical College, New Taipei City, Taiwan

**Keywords:** food away from home (FAFH), gastrointestinal, health, consumption behavior, dietary behavior

## Abstract

Dietary behavior is a critical lifestyle factor affecting health. This study aimed to investigate food away from home (FAFH) and its effect on gastrointestinal (GI) health. A questionnaire-based survey was conducted with 300 participants at a hospital in Liouying, Taiwan. The survey collected demographic information and data on FAFH and GI health. The association of GI health with FAFH consumption behavior was significant (*t*-test, *p* < 0.05). Bodyweight status was associated with age (*F* = 5.01, *p* = 0.01), dietary situation (*F* = 1.96, *p* = 0.04), number of meals (*F* = 1.85, *p* = 0.03), dietary preferences (*F* = 2.84, *p* = 0), reasons for FAFH (*F* = 1.86, *p* = 0.02), FAFH types (*F* = 2.01, *p* = 0), and outcomes associated with FAFH (*F* = 2.51, *p* = 0). Gastrointestinal condition was associated with the number of meals (*F* = 2.55, *p* = 0), the level of activity after meals (*F* = 2.16, *p* = 0.02), and FAFH type (*F* = 1.48, *p* = 0.04). The results indicated that the participants aged 20–40 years had more problems related to their self-perceived body weight status than those aged 41–50 years. The results of this study clarify the FAFH among people in Taiwan and the effects on GI health and may serve as a reference for relevant behavioral research in food and health studies.

## Introduction

Economic development and changes in lifestyle and diseases have an impact on health demands (1, 2); thus, investigating the relationship between dietary behavior as one major component of lifestyle and human health is critical. Economic development and lifestyle changes have also led to an increasing number of people consuming food away from home (FAFH) (3–5). Clarifying the effect of health-related factors, such as dietary behavior, on gastrointestinal (GI) health is paramount (6, 7). Dietary behavior is a major lifestyle factor affecting health (8–12); relevant studies have reported that nutritional knowledge affects the lifestyle of people and may even cause health risks because of the correlation between dietary behaviors and lifestyle (13–16). However, studies on the impact of FAFH and its effect on GI health are relatively scant; thus, this topic merits further research.

Unhealthy dietary habits are a major cause of disease in individuals of all ages (17, 18). Food away from home has been reported to shift with social changes and dietary development, particularly among those aged >50 years (4, 10). Furthermore, researchers have suggested that awareness of healthy diets with respect to FAFH should be promoted among older people because of the health needs of an aging population (4, 10). A study of people aged >50 years regarding eating-out behavior in Taiwan did not identify an association between FAFH and self-perceived GI health status (4). The researchers attributed this finding to the tendency among the participants to avoid consuming salty and spicy FAFH and to limit their overall FAFH consumption. The study did not observe an association between GI symptoms, such as abdominal pain, GI discomfort, constipation, and FAFH consumption behaviors (4). Therefore, the age factor should receive more attention when considering food consumption behaviors and whether they are associated with GI health.

Studies have also indicated that sex differences may affect health behaviors related to diet (18, 19). A study of FAFH consumption among Korean adolescents (18) revealed that adolescents favored spicy and salty foods, which is indicative of fast-food and processed food consumption. Another study indicated that Iranian female adolescents had high scores for snacking; their eating patterns were also associated with more mental health problems (19).

Several other international studies have revealed that food (20–24), diet (25–27), diet quality (28, 29), and eating behaviors (30) are associated not only with obesity and health problems but also with the quality of life (31–35) and related life satisfaction (36). The effects of FAFH on GI health merit greater attention. In this study, we explored FAFH and its effect on GI health.

## Materials and Methods

### Study Design and Setting

A questionnaire-based survey was conducted with 300 participants aged 20–80 years at the Chi Mei Medical Center (Liouying District, Tainan City, Taiwan) from July 2016 to June 2017. The sample size had power of 0.8 with an alpha significance of 0.05 and an effect size of 0.8 (37). The study participants were required to speak Mandarin or Taiwanese Hoklo dialect and to have no cognitive disorders that could affect their participation. The study was conducted in accordance with the STROBE (Equator Guidelines). All the participants provided written informed consent.

**Figure 1:**
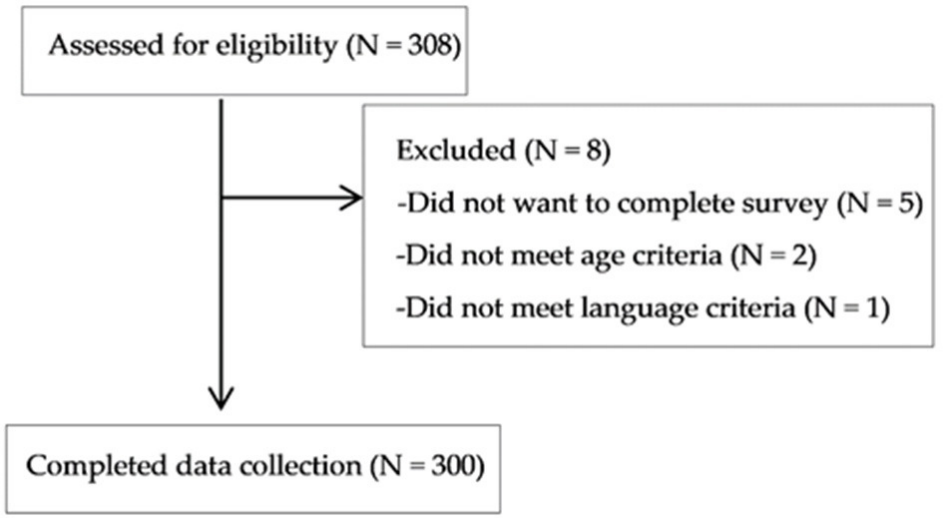
A flowchart of participant recruitment.

A flowchart of participant recruitment is provided in Figure 1. We assessed 308 participants for eligibility, and 8 participants were excluded: 5 declined to complete the survey, 2 did not meet the age criterion, and 1 did not meet the language criterion. Therefore, we completed the data collection in 300 participants.

### Ethical Considerations

The study was conducted in accordance with the Declaration of Helsinki and approved by the regional ethics committee of the Institutional Review Board of Chi Mei Medical Center, Tainan, Taiwan (IRB Serial No: 10406-L01).

### Measurements

The survey was a self-reported structured questionnaire (38) to collect demographic data and to assess the FAFH and GI health of respondents. Demographic information was collected on age, sex, ethnicity, education level, marital status, and religion. The questionnaire on FAFH contained 40 questions in eight categories: dietary situation, number of meals, dining situation, level of activity after meals, dietary preferences, the reason for FAFH, FAFH types, and outcomes associated with FAFH (38). Answers were provided on a five-point scale (0, *never*; 1, *rarely*; 2, *occasionally*; 3, *often*; and 4, *always*) (38). The higher the score means the seriousness of FAFH behavior. The Cronbach's α for the questionnaire was 0.85, revealing favorable internal consistency (38).

The dietary situation was assessed with three questions as eating habits, on whether the participants regularly consumed breakfast, lunch, and dinner. For the number of meals that the participants consumed per day, five questions were employed (one, two, three, four, or five or more meals per day).

The dining situation was assessed with four questions about religious or medical food restrictions, perceived comfort with the dining environment, the experience of pain and discomfort during meals, and consumption of nutritional food supplements (38).

For the level of activity after meals, the participants rated three items: working, resting, or walking. For dietary preferences, the participants rated five items: salty, spicy, sour, sweet, or plain foods. The reasons for FAFH included five questions related to family composition, which family members were involved in food preparation and the availability and convenience of various FAFH options. Preferred FAFH types of participants were assessed through nine questions. The FAFH types included vegetarian cuisine, Japanese cuisine, Chinese cuisine, Western cuisine, local cuisine, foods from quick-service restaurants, casual dining, food from buffet restaurants, and food from night markets. Finally, the survey also included six questions on outcomes associated with FAFH, such as frequency of FAFH [e.g., occasional, almost daily, during weekends and public holidays, during visits to relatives and friends, and during travel or special anniversaries; (38)].

The assessment of GI health included two items: body weight status and GI condition. Bodyweight status included five questions: self-perceived assessment of being overweight, being underweight, having abdominal obesity (e.g., potbelly and beer belly), having lipedema (accumulation of fat cells in the tissues under the skin), and being obese. The self-perceived assessment of GI condition included 11 questions about gastroesophageal reflux disease (GERD), heartburn, nausea, dysphagia, swallowing difficulties, hyperchlorhydria, hypochlorhydria (a low level of stomach acid), duodenal ulcer, food absorption problems, colon-related complications, and constipation. Answers were provided on a five-point scale (0, *never*; 1, *rarely*; 2, *occasionally*; 3, *often*; and 4, *always*) (38). The higher the score, the more problems related to their self-perceived GI health. The Cronbach's α for the questionnaire was 0.84, revealing favorable internal consistency (38).

### Data Analyses

All statistical analyses were performed using SPSS version 22.0 (SPSS Inc., Chicago, IL, USA). The data were analyzed using frequencies and percentages, *t*-tests, and Scheffe's test. The resulting content validity index was 0.85, confirming that the questionnaire was appropriate and applicable. The reliability of our research was assessed using Cronbach's α to assess internal consistency. Cronbach's α ranged from 0.84 to 0.85. For all statistical tests, *p* < 0.05 indicated significance.

## Results

### Participant Demographics

The participants had a mean age of 41 years (SD: 12 years). Most of the participants (63%) were aged 20–40 years, and they were more likely to be women (*n* = 221, 73.7%), Hoklo Taiwanese (*n* = 274, 91.3%), college or university graduates (*n* = 181, 60.3%), and married (*n* = 186, 62%); the most common religion was Taoism (*n* = 129, 43%; Table 1).

**Table 1:** Participant demographics (*N* = 300).

**Variable**		***N***	**%**	
Age				Mean age (SD): 41.16 (12.46)
	20–40 years	189	63	
	41–50 years	45	15	
	51–64 years	46	15.3	
	65–80 years	20	6.7	
**Gender**				
	Male	79	26.3	
	Female	221	73.7	
**Ethnicity**				
	Hoklo Taiwanese	274	91.3	
	Chinese	13	4.4	
	Hakka	7	2.3	
	Aborigine and other	6	2.0	
**Education**				
	None	3	1	
	Primary school	23	7.7	
	Junior high school	14	4.7	
	High school	55	18.3	
	College/university	181	60.3	
	Research institute	24	8	
**Marriage**				
	Married	186	62	
	Separated	3	1	
	Single	100	33.3	
	Widowed	11	3.7	
**Religion**				
	Buddhist	65	21.7	
	Taoist	129	43	
	Christian	12	4	
	None	92	30.6	
	Other	2	0.7	

*SD, standard deviation*.

### Assessment of FAFH

The results of the survey showed that dietary situation, number of meals, dining situation, level of activity after meals, dietary preferences, reason of FAFH, FAFH types, and outcomes associated with FAFH were significantly different among the participants (Table 2).

**Table 2:** FAFH consumption behavior scores of the participants (*N* = 300).

**Items**	**Mean ± SD**	***t***	***p*** **-value**
Dietary situation	9.98 ± 2.06	83.54	<0.05*
Number of meals	5.98 ± 2.62	39.46	<0.05*
Dining situation	5.23 ± 2.58	35.08	<0.05*
Level of activity after meals	5.85 ± 1.69	59.94	<0.05*
Dietary preferences	6.74 ± 3.08	37.84	<0.05*
Reason for FAFH	8.40 ± 4.66	31.19	<0.05*
FAFH types	18.22 ± 6.40	49.26	<0.05*
Outcomes associated with FAFH	13.09 ± 5.26	43.04	<0.05*

*FAFH, food away from home*.

* *Significant at p < 0.05*.

### Assessment of GI Health

**Figure 2:**
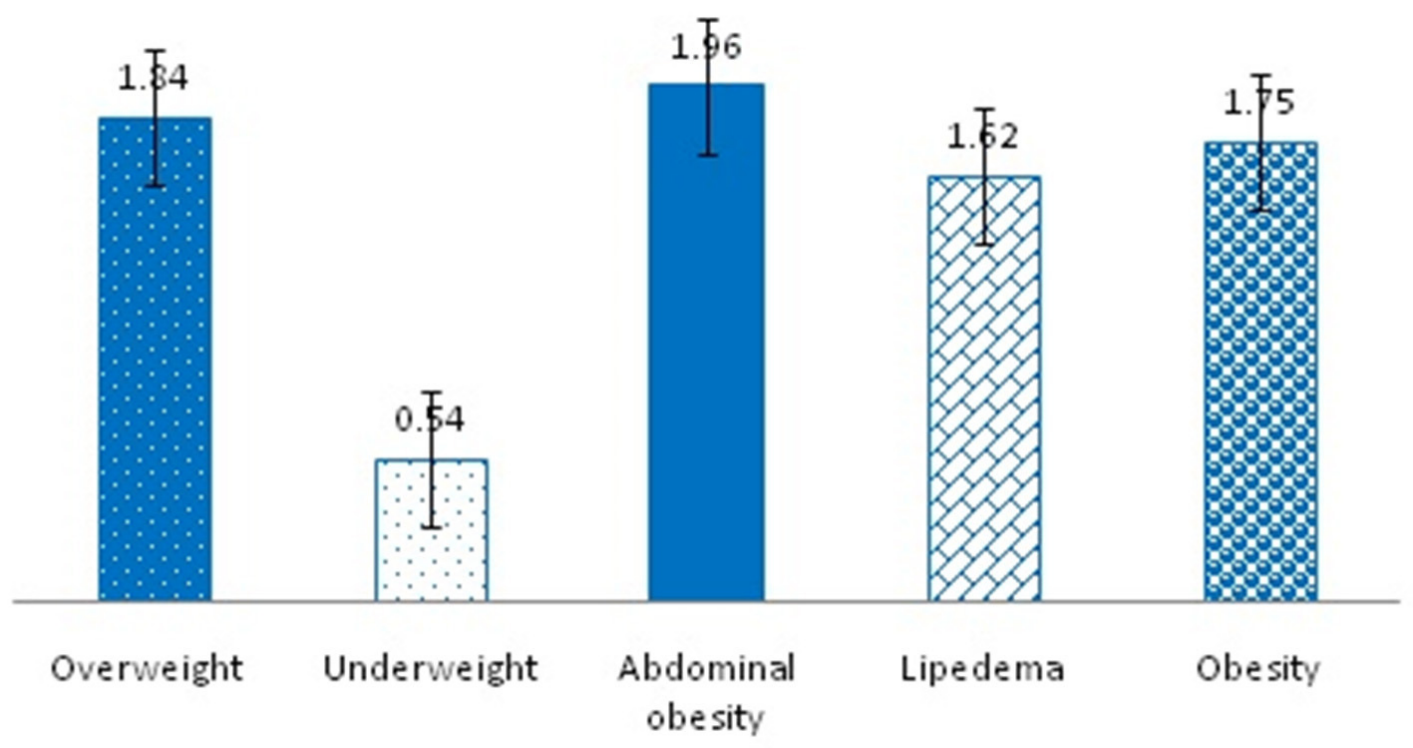
Self-perceived in the bodyweight status.

Self-perceived abdominal obesity (e.g., having a potbelly or a beer belly) had the highest score (1.96 ± 1.34) in the bodyweight status category, followed by being overweight (1.84 ± 1.36), being obese (1.75 ± 1.39), having lipedema (1.62 ± 1.28), and being underweight (0.54 ± 0.89) (Table 3; Figure 2).

**Table 3:** Predictors of GI health of the participants (*N* = 300).

**Items**	**Mean ± SD**	***t***	***p*** **-value**
Body weight status	7.71 ± 4.84	27.59	<0.05*
Overweight	1.84 ± 1.36		
Underweight	0.54 ± 0.89		
Abdominal obesity (potbelly/beer belly)	1.96 ± 1.34		
Lipedema	1.62 ± 1.28		
Obesity	1.75 ± 1.39		
GI condition	8.46 ± 6.45	22.69	<0.05*
GERD	1.35 ± 1.12		
Heartburn	0.94 ± 1.01		
Nausea	1.20 ± 1.07		
Dysphagia	0.45 ± 0.67		
Swallowing difficulties	0.38 ± 0.61		
Hyperchlorhydria	1.22 ± 1.10		
Hypochlorhydria	0.37 ± 0.59		
Duodenal ulcer	0.46 ± 0.81		
Food absorption problems	0.39 ± 0.63		
Colon-related complications	0.58 ± 0.87		
Constipation	1.07 ± 1.14		

*Data are presented as mean ± standard deviation (SD); GI, gastrointestinal; GERD, gastroesophageal reflux disease*.

* *Significant at p < 0.05*.

**Figure 3:**
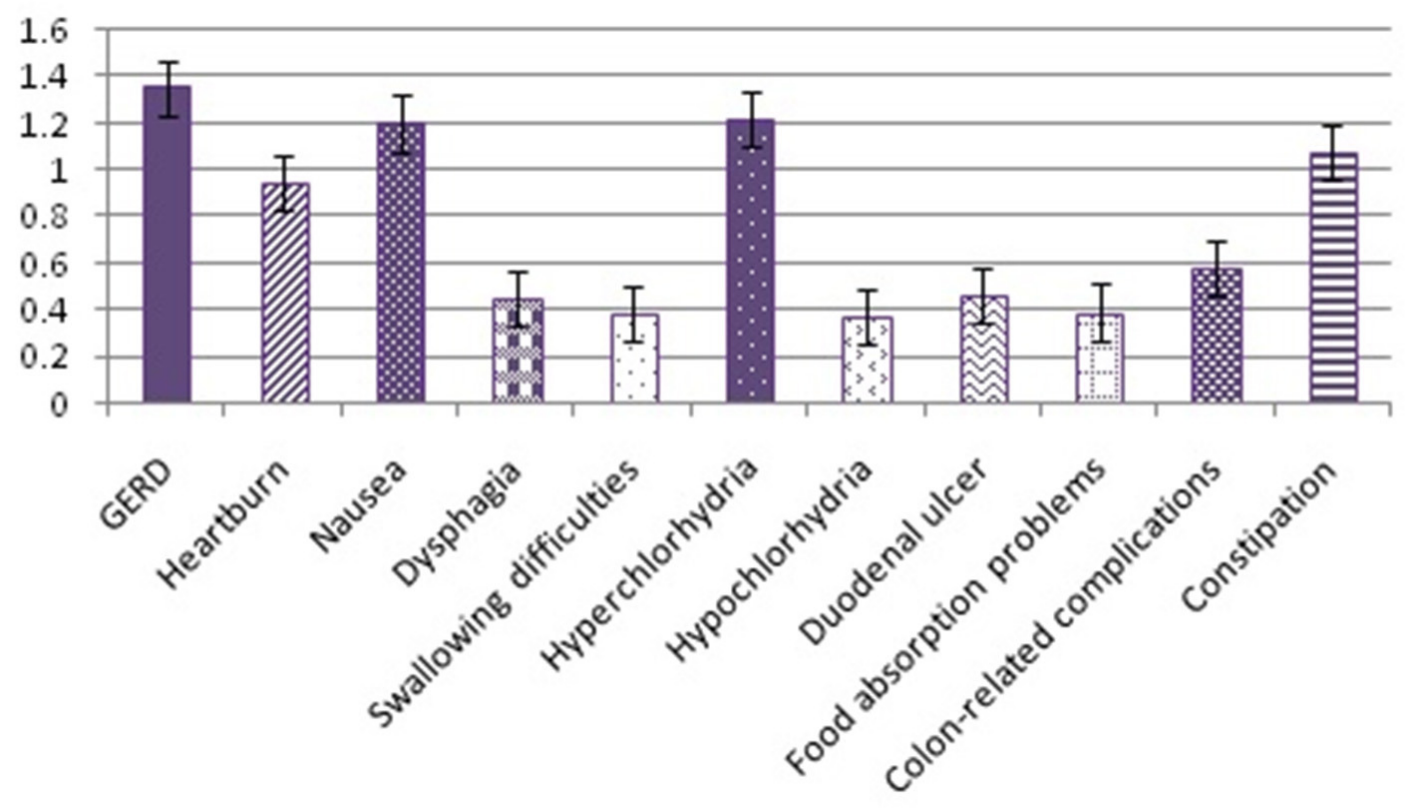
Self-perceived in the gastrointestinal (GI) condition.

Self-perceived GERD had the highest score (1.35 ± 1.12) in the GI condition category, followed by hyperchlorhydria (1.22 ± 1.1), nausea (1.20 ± 1.07), constipation (1.07 ± 1.14), heartburn (0.94 ± 1.01), colon-related complications (0.58 ± 0.87), duodenal ulcer (0.46 ± 0.81), dysphagia (0.45 ± 0.67), food absorption problems (0.39 ± 0.63), swallowing difficulties (0.38 ± 0.61), and hypochlorhydria (0.37 ± 0.59) (Table 3; Figure 3).

Both body weight status and GI condition were significant predictors of GI health (Table 3).

### Predictors of GI Health

The predictors of GI health are listed in Table 4. Subsequent comparisons using Scheffe's *post-hoc* analysis also revealed significant differences (1>2); an age factor of 1, 2, 3, and 4 represented those aged 20–40 years, 41–50 years, 51–64 years, and 65–80 years, respectively. The results indicated that the participants aged 20–40 years had more problems related to their self-perceived body weight status than those aged 41–50 years.

**Table 4:** Predictors of GI health (*N* = 300).

**Predictor**	**GI health**				
	**Bodyweight status**			**GI condition**	
	***F***	***p*** **-value**	**(Scheffe's test)**	***F***	***p*** **-value**
Age	5.01	0.01*	(1>2)^a^	1.12	0.34
Gender	3.17	0.08		1.83	0.18
Ethnicity	1.40	0.24		2.60	0.05
Education	0.90	0.48		0.79	0.55
Marriage	1.97	0.12		0.94	0.42
Religion	0.18	0.94		1.39	0.24

*GI, gastrointestinal; Scheffe's test, Scheffe's post-hoc analysis*.

a *Scheffe's post-hoc analysis was conducted as follows: an age factor of 1 represents 20–40 years, 2 represents 41–50 years, 3 represents 51–64 years, and 4 represents 65–80 years*.

* *Significant at p < 0.05*.

### FAFH and GI Health

Food away from home is presented in Table 5. Significant differences were observed in the dietary situation, the number of meals, dietary preferences, reasons for FAFH, FAFH types, and outcomes associated with FAFH in relation to body weight status (*p* < 0.05). Significant differences were also noted in the number of meals, level of activity after meals, and FAFH types in relation to GI condition (*p* < 0.05).

**Table 5:** Association of GI health with FAFH consumption behavior (*N* = 300).

**FAFH consumption behavior**	**GI health**			
	**Bodyweight status**		**GI condition**	
	***F***	***p*** **-value**	***F***	***p*** **-value**
Dietary situation	1.96	0.04*	1.75	0.08
Number of meals	1.85	0.03*	2.55	0.00*
Dining situation	1.21	0.27	1.57	0.09
Level of activity after meals	1.00	0.44	2.16	0.02*
Dietary preferences	2.84	0.00*	1.47	0.12
Reason for FAFH	1.86	0.02*	1.54	0.07
FAFH types	2.01	0.00*	1.48	0.04*
Outcomes associated with FAFH	2.51	0.00*	1.20	0.24

*FAFH, food away from home; GI, gastrointestinal*.

* *Significant at p < 0.05*.

## Discussion

This study investigated FAFH and its effect on GI health among people in a single-center hospital setting in Taiwan. Significant differences were observed in all eight measures used to measure FAFH. These results are consistent with those of previous studies. The relationship between diet and health is associated with changes in lifestyle patterns (3–5, 39, 40); thus, attention should be paid to the increase in FAFH that accompanies economic development and lifestyle changes.

The significant differences in body weight status and GI condition are also consistent with the results of previous studies (8, 9, 14, 16). Further research is warranted to clarify how GI health status affects overall health needs. The association between age and body weight status was also consistent with the findings of previous studies (3, 4, 6, 7). Given that the FAFH of adults also changes because of social changes and economic development, adopting healthy dietary habits to maintain GI health should be emphasized. Notably, although no significant relation was observed between gender and body weight, the finding almost reached significance and is consistent with the finding of a related study (41). The influence of sex and body weight status on FAFH merits further research.

This study suggested that healthcare providers and professionals should prioritize dietary recommendations and health topics according to the health behaviors of individuals and should adopt health education according to their ages. For example, individuals aged 20–40 years might benefit from food education on body weight control, FAFH, and GI health. Developing more effective education strategies by adding new topics (e.g., major food safety concerns, behavioral nutrition, dietary prevention of GI disease, and dietary behavior screening) to enable in their daily lives implement correctly eating behavior to improve the overall GI health of the individual.

The results of this study address a gap in knowledge about the effect of FAFH on GI health in a single-center hospital setting in Taiwan. However, this study has some limitations. First, this study has not asked participants their primary reasons for coming to the hospital. This, obviously, might have put a major bias, such as the participants were referred or they came to the hospital only for their actual/self-perceived gastrointestinal problems, which could directly interfere with the main outcome of this study, i.e., it might be possible that to overestimate the score of participants with self-perceived GI health status. Second, this study used a self-reported survey to explore FAFH and GI health; thus, responses may not be a full expression of the actual lifestyles of the participants. Third, this study was conducted at a single center in Taiwan; generalizing the results to other populations should, therefore, be undertaken with caution. In particular, some of the questions addressed sensitive topics, such as the level of activity after meals and reasons for FAFH. The sensitivity of these topics may have led to a reporting bias; it may need a further deep interview or discussion. Last, this study has collected some data on marital status, religion, and gender but did not explore the association with FAFH; it might be intuitively logical to think that marital (being married) and employment status (being unemployed or both husband and wife are employed, etc.) and the gender of the spouse of the participants might affect dietary behaviors, such as FAFH. Consequently, these might have affected their willingness to respond correctly/honestly. Hence, as a suggestion for future studies, such factors must be considered in further designs.

The current results revealed a significant association between GI health and the FAFH of the study participants. In particular, significant differences were noted in dietary situations, the number of meals, dietary preferences, the reason for FAFH, FAFH types, and outcomes associated with FAFH according to body weight status. Significant differences were also observed in the number of meals, level of activity after meals, and FAFH types according to GI condition. Collectively, these results indicate that FAFH can associate with GI health and should thus be further explored. The current results expand the knowledge of the relationship between FAFH and GI health and, thus, may serve as a reference for relevant behavioral research in food and health studies.

## Data Availability Statement

The raw data supporting the conclusions of this article will be made available by the authors, without undue reservation.

## Ethics Statement

The studies involving human participants were reviewed and approved by the Regional Ethics Committee of the Institutional Review Board of Chi Mei Medical Center, Tainan, Taiwan (IRB Serial No: 10406-L01). Written informed consent for participation was not required for this study in accordance with the national legislation and the institutional requirements.

## Author Contributions

J-JC and C-YH: conceptualization, formal analysis, resources, and writing original draft preparation. C-YH: methodology, software, data curation, writing—review and editing, visualization, supervision, project administration, and funding acquisition. J-JC, L-YT, J-MT, and C-YH: validation. J-JC: investigation. All authors contributed to the article and approved the submitted version.

## Funding

This research was supported by the Chi Mei Medical Center Research Fund (Grant No. 10406-L01).

## Conflict of Interest

The authors declare that the research was conducted in the absence of any commercial or financial relationships that could be construed as a potential conflict of interest.

## Publisher's Note

All claims expressed in this article are solely those of the authors and do not necessarily represent those of their affiliated organizations, or those of the publisher, the editors and the reviewers. Any product that may be evaluated in this article, or claim that may be made by its manufacturer, is not guaranteed or endorsed by the publisher.
